# The complete chloroplast genome sequence of *Acorus tatarinowii* (Araceae) from Fujian, China

**DOI:** 10.1080/23802359.2020.1806133

**Published:** 2020-08-12

**Authors:** Liang Ma, Shu-Zhen Jiang, Hui Lian, Yuan-Fang Xiong, Zhong-Jian Liu, Shi-Pin Chen

**Affiliations:** a College of Forestry, Fujian Agriculture and Forestry University, Fuzhou, China; b Key Laboratory of National Forestry and Grassland Administration for Orchid Conservation and Utilization at College of Forestry, Fujian Agriculture and Forestry University, Fuzhou, China; c College of Landscape, Fujian Agriculture and Forestry University, Fuzhou, China

**Keywords:** *Acorus tatarinowii*, chloroplast genome, phylogeny, Araceae

## Abstract

*Acorus tatarinowii* is a useful traditional Chinese medicine (TCM) and is officially documented in the Chinese Pharmacopeia with the name ‘Shi Chang Pu’. It belongs to the Araceae family and is used for the treatment of dementia, epilepsy, amnesia and insomnia. We resequenced complete chloroplast (cp) genome of *A. tatarinowii* from Fujian, China. The whole genome was 153,453 bp in length, consisting of a pair of inverted repeats (IR 25,795 bp), a large single-copy region (LSC 83,631 bp), and a small single-copy region (SSC 18,232 bp). The complete genome contained 132 genes, including 84 protein-coding genes, 38 tRNA, and 8 rRNA genes. The overall GC content of the whole genome was 38.7%. A maximum-likelihood phylogenetic analysis showed that *A. tatarinowii* is sister to *A. tatarinowii* which was collected in Yunnan, China. The complete chloroplast genome of *A. tatarinowii* will help improve and integrate the existing genome data of monocots and provide insights into the phylogenetic relationship among basal angiosperms, monocots and dicots.

*Acorus tatarinowii* is a multi-year-old herbaceous plant of the Araceae family (Zhang et al. 2015; Sun 2020); the whole grass is similar to gramineous, with green pedicels, leaf buds, and aromatic rhizomes. The rhizomes have been used as medicine for thousands of years (Wu 2017). *A. tatarinowii* is mainly distributed in various provinces in the south of China’s Yellow River Basin. The current main producing area is Sichuan, and Fujian Province has a large reserve. Previously, a cp genome of *A. tatarinowii* has been reported (Gong et al. 2020). To compare the chloroplast of *A. tatarinowii* from different distributed regions, we sequenced the complete chloroplast genome of *A. tatarinowii* from Fujian, China.

Leaf sample of *A. tatarinowii* was collected from Tianmen Mountain, Fujian Province, China (25°49′18.78″N, 119°1′6.90″E), and dried into silica gel immediately. The voucher specimen is kept at the Hherbarium of College of Forestry, Fujian Agriculture and Forestry University (Voucher specimen: FAFU1190428006).

DNA was extracted from the fresh leaf tissue, with 500 bp randomly interrupted by the Covaris ultrasonic breaker for library construction. The constructed library was sequenced PE150 by Illumina Hiseq Xten platform, approximately 5GB data were generated. Illumina data were filtered by script in the cluster (default parameter: −L 5, −p 0.5, −N 0.1). Complete chloroplast genome of *A. tatarinowii* (GeneBank accession: NC_045294) was used as reference, chloroplast genome of *A. tatarinowii* was assembled by Get Organelle pipe-line (https://github.com/Kinggerm/GetOrganelle). It can get the plastid-like reads, and the reads were viewed and edited by Bandage (Wick et al. 2015). Assembled chloroplast genome annotation were based on comparison with *C. echinocarpa* by Geneious v 11.1.5 (Biomatters Ltd., Auckland, New Zealand) (Kearse et al. 2012). The annotation result was draw with the online tool OGDRAW (http://ogdraw.mpimp-golm.mpg.de/) (Lohse et al. 2013; Jiang et al. 2019).

The complete chloroplast genome sequence of *A. tatarinowii* (GeneBank accession: MT755635) was 153,453 bp in length, with a large single-copy (LSC) region of 83,631bp, a small single-copy (SSC) region of 18,960 bp, and a pair of inverted repeats (IR) regions of 25,795 bp. Complete chloroplast genome contain 132 genes, there were 84 protein-coding genes, 38 tRNA genes, and 8 rRNA genes. The complete genome GC content was 38.7%.

To investigate the phylogenetic position of *A. tatarinowii*, 24 complete cp genomes of species from Poaceae, Commelinids, Lilianae, Dioscoreales, Pandanales, Liliales, Chloranthaceae, Petrosaviales, Lemnaceae, Araceae, Alismatales, Acoranae, Lauraceae, Calycanthaceae, were downloaded from NCBI and were aligned using MAFFT v7.307 (Katoh and Standley 2013). RAxML (Stamatakis 2014) was used to construct a maximum likelihood tree with *Litsea glutinosa* and *Calycanthus chinensis* as outgroup. The branch support was computed with 1000 bootstrap replicates. The ML tree analysis indicated that *A. tatarinowii* and *A. tatarinowii* (NC_045294) cluster together with 100% bootstrap support (Figure 1).

**Figure 1.**
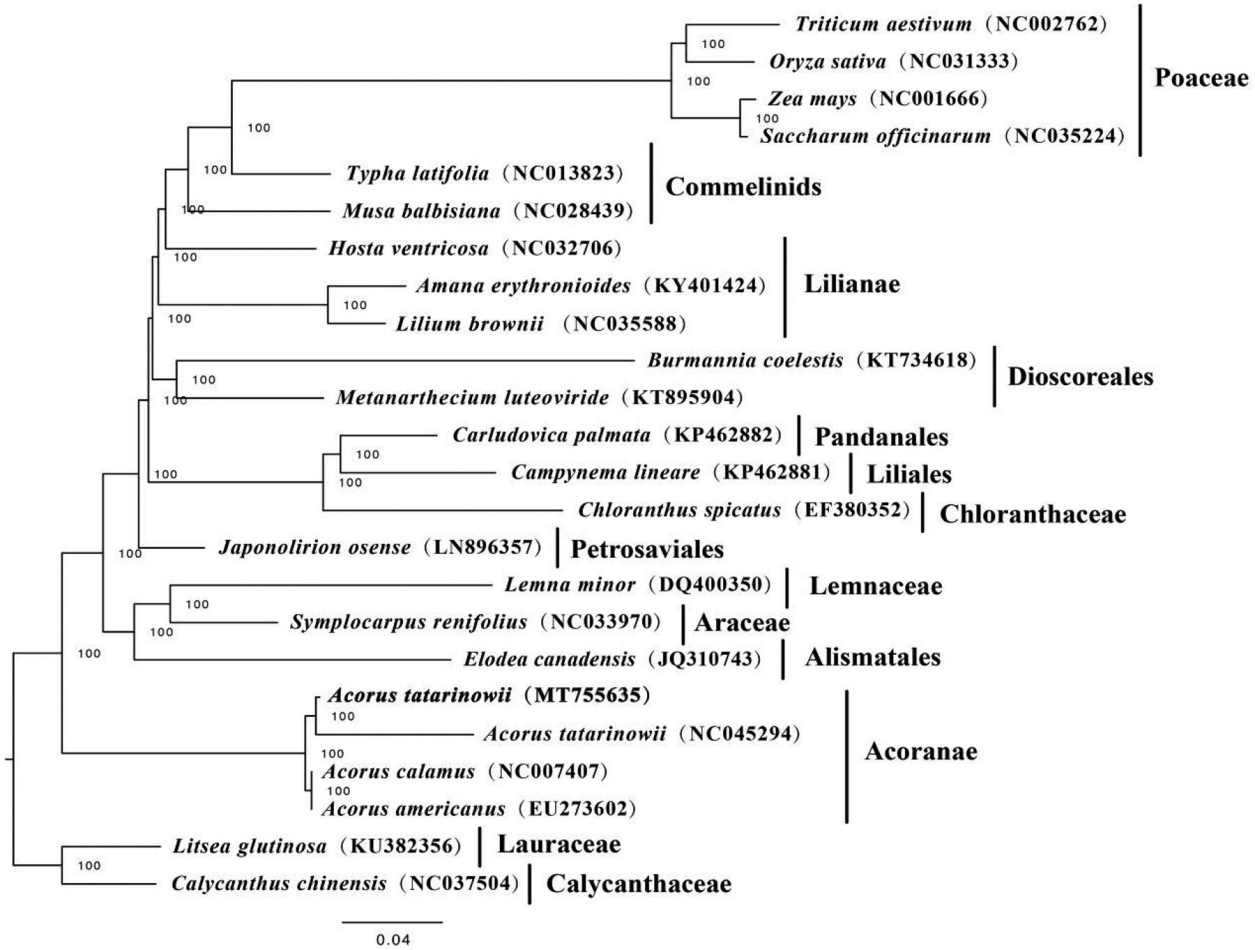
Maximum likelihood tree based on the complete chloroplast genome sequences of 24 species, with *Litsea glutinosa* and *Calycanthus chinensis* as outgroup. The bootstrap value is shown on each node and the position of *Acorus tatarinowii* is in bold.

## Data Availability

The data that support the findings of this study are available in [NCBI] at [https://www.ncbi.nlm.nih.gov/], reference number [MT755635].
